# Benzyl 2-β-Glucopyranosyloxybenzoate, a New Phenolic Acid Glycoside from *Sarcandra glabra*

**DOI:** 10.3390/molecules17055212

**Published:** 2012-05-04

**Authors:** Haifeng Wu, Xiaoru Hu, Xiaopo Zhang, Shilin Chen, Junshan Yang, Xudong Xu

**Affiliations:** 1 Institute of Medicinal Plant Development, Chinese Academy of Medical Sciences & Peking Union Medical College, Beijing 100193, China; Email: wwwtony505@yahoo.com.cn (H.W.); huxru2008@126.com (X.H.); xiaopozhang2011@126.com (X.Z.); slchen@implad.ac.cn (S.C.); junshanyang@sina.com (J.Y.); 2 National Institutes for Food and Drug Control, Beijing 100050, China

**Keywords:** *Sarcandra glabra*, chloranthaceae, benzyl 2-β-D-glucopyranosyloxybenzoate, anticancer activity

## Abstract

From the whole plant of *Sarcandra glabra*, a new phenolic acid glycoside, benzyl 2-β-glucopyranosyloxybenzoate (**1**), together with seven known compounds including eleutheroside B_1_ (**2**), 5-*O*-caffeoylshikimic acid (**3**), (–)-(7*S*, 8*R*)-dihydrodehydro-diconiferyl alcohol (**4**), (–)-(7*S*, 8*R*)-dihydrodehydrodiconiferyl alcohol 9-, 9′- and 4-*O*-â-D-glucopyranoside (**5**–**7**), and (–)-(7*S*, 8*R*)-5-methoxydihydrodehydrodiconiferyl alcohol 4-*O*-β-D-glucopyranoside (**8**) was isolated. Their structures were elucidated by spectral analysis including 1D-, 2D-NMR and HR-ESI-MS. Compound **2** was found to exhibit potent cytotoxic activity against BGC-823 and A2780 cancer cell lines using MTT method with IC_50_ value of 2.53 and 1.85 µM, respectively.

## 1. Introduction

**Figure 1:**
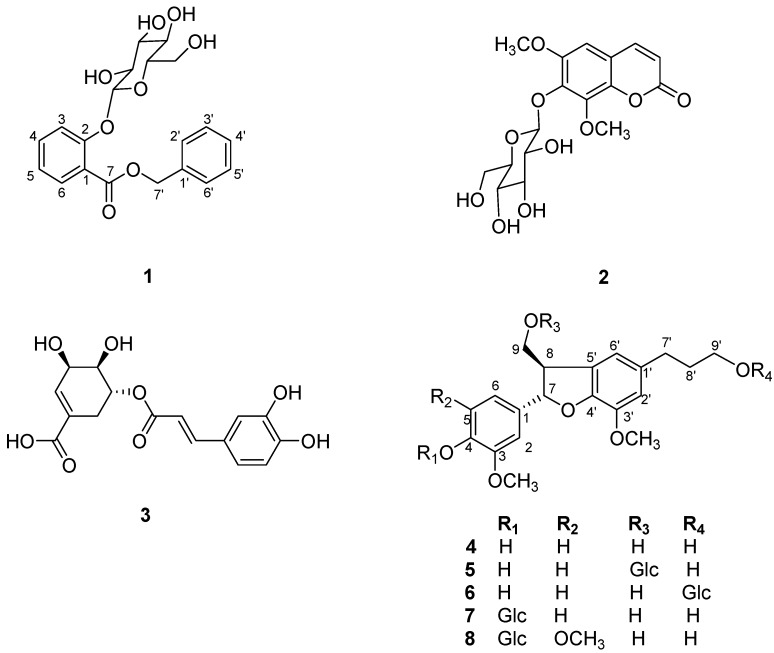
Structures of compounds **1**–**8**.

*Sarcandra glabra* (Thunb.) Makino (Chloranthaceae), an evergreen shrub growing in southern China, has been used in traditional medicine to treat bruises, bone fractures and arthritis [1]. The whole plant of *S. glabra* is specified in the 2010 edition of the Chinese Pharmacopoeia as a traditional medicine used for its anticancer, antibacterial and antivirus activities [2]. So far, there have been many reports on the constituents in the whole plant of *S. glabra*, demonstrating the presence of sesquiterpenoids, coumarins, flavonoids, triterpenoids and phenolic acids [1,3,4,5,6,7,8,9,10,11,12,13,14,15,16,17,18]. Our previous phytochemical studies on the species resulted in the isolation of a new coumarin and three novel sesquiterpene glycosides [19,20]. In the continuation of our efforts to investigate antitumor agents from this plant, a new phenolic acid glycoside, benzyl 2-β-glucopyranosyloxybenzoate (**1**) was isolated and determined using MS and NMR techniques. Also isolated from the 70% acetonic extract of the whole plants of *S. glabra *are eleutheroside B_1_ (**2**) [21], 5-*O*-caffeoylshikimic acid (**3**) [17], (–)-(7*S*, 8*R*)-dihydrodehydrodiconiferyl alcohol (**4**) [22], (–)-(7*S*, 8*R*)-dihydrodehydrodiconiferyl alcohol 9-, 9′- and 4-*O*-â-D-glucopyranoside (**5**–**7**) [22], and (–)-(7*S*, 8*R*)-5-methoxy-dihydrodehydrodiconiferyl alcohol 4-*O*-β-D-glucopyranoside (**8**) [22] (Figure 1). In this paper, we report the isolation and structural elucidation of the new compound, as well as cytotoxicities of compound **2** against human cancer cell lines.

## 2. Results and Discussion

Compound **1** was obtained as a colorless amorphous powder and gave positive test with Molish reagent, thereby indicating its glycosidic nature. The HR-ESI-MS of **1** showed a quasi-molecular ion peak at *m/z* 413.1192 [M + Na]^+^ (calcd. 413.1212 [M + Na]^+^) in the positive-ion mode. In conjunction with the analysis of ^1^H- and ^13^C-NMR spectra, the molecular formula of **1** was deduced as C_20_H_22_O_8_, with 10 degrees of unsaturation. The IR spectrum showed hydroxyl groups (3,383 cm^−1^), an ester group (1,706 cm^−1^) and an aromatic ring (1601, 1490 and 1074 cm^−1^). The ^1^H-NMR spectrum (Table 1) indicated the presence of 1,2-disubstituted benzene ring, *δ*_H_ 7.76 (1H, dd, *J* = 8.4, 1.8 Hz), 7.54 (1H, m), 7.39 (1H, dd, *J* = 7.8, 1.8 Hz) and 7.13 (1H, m), a monosubstituted benzene ring, *δ*_H_ 7.45 (2H, d, *J* = 7.2 Hz), 7.39 (2H, t, *J* = 7.8 Hz) and 7.33 (1H, m), an oxymethylene group (*δ*_H_ 5.33) and an anomeric proton (*δ*_H_ 4.92). The remaining sugar protons resonated from *δ*_H_ 3.91 to 3.39. The ^13^C-NMR spectrum (Table 1) displayed 20 signals for ester carbon at *δ*_C_ 168.2 (C-7), aromatic carbons from *δ*_C_ 158.8 to 119.0, anomeric carbon at *δ*_C_ 104.0 (C-10), other sugar carbons between *δ*_C_ 78.6 and 62.7 and oxygenated methylene carbon at *δ*_C_ 68.2. The HMBC spectrum of **1** showed correlations of C-7 with H-7′ and H-6, C-2 with H-3, C-7′ with H-2′ and 6′, C-1′ with H-7′, C-1 with H-3, C-3 with H-4, C-4 with H-5, and C-6 with H-5. The sugar group was presented at C-2, as indicated from the HMBC correlation between the anomeric proton (*δ*_H_ 4.92) and C-2 (*δ*_C_ 158.8). The ^1^H- and ^13^C-NMR data of **1** was completely assigned with the help of HMBC and HSQC, and presented in Table 1.

**Table 1:** ^1^H- and ^13^C-NMR data of compound **1 **in CD_3_OD (δ in ppm, *J *in Hz).

Position	*δ*_C_	*δ*_H_	HMBC (H to C)
1	122.6		
2	158.8		
3	119.0	7.39 (dd, 7.8, 1.8)	C-1, C-2
4	135.4	7.54 (m)	C-3
5	123.9	7.13 (m)	C-4, C-6
6	132.3	7.76 (dd, 8.4, 1.8)	C-7
7	168.2		
1′	137.5		
2′, 6′	129.4	7.45 (d, 7.2)	C-1′
3′, 5′	129.8	7.39 (m)	
4′	129.5	7.33 (m)	
7′	68.2	5.34 (dd, 12, 12)	C-7, C-1′
Glc-1	104.0	4.92 (d, 7.2)	C-2
2	75.1	3.52 (m)	
3	77.7	3.48 (m)	
4	71.4	3.40 (m)	
5	78.6	3.46 (m)	
6	62.7	3.71 (dd, 12.0, 6.0)	
		3.89 (dd, 12.0, 1.8)	

**Figure 2:**
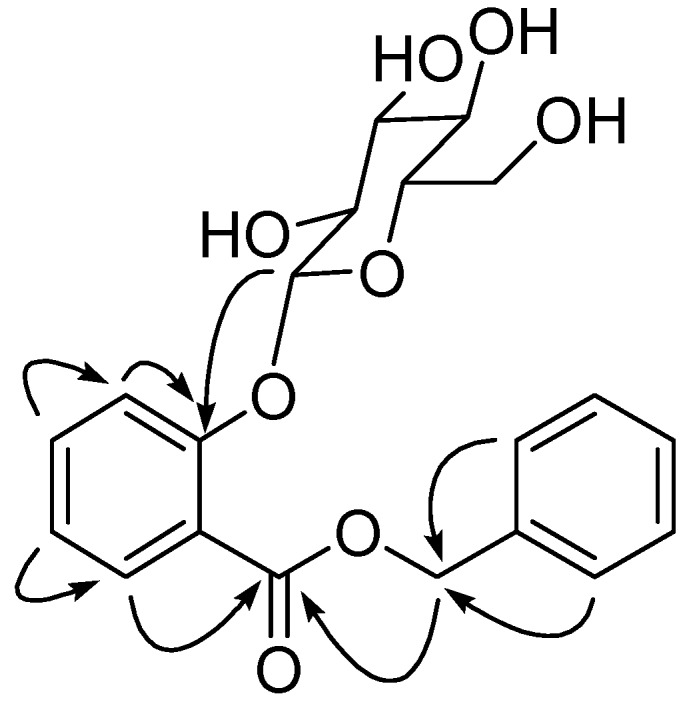
Key HMBC correlations of 1.

The key HMBC correlations of **1** are shown in Figure 2. The absolute configuration of the glucose was not determined. On the basis of the above observations, compound **1** was assigned as benzyl 2-β-glucopyranosyloxybenzoate. This is a new compound. The isolated compound **2** was evaluated using the MTT method *in vitro* for cytotoxic activities against five human cancer cell lines, HCT-8 (colon cancer), Bel-7402 (liver cancer), A549 (lung carcinoma), BGC-823 (gastric cancer) and A2780 (ovarian cancer). The results showed that the IC_50_ values of eleutheroside B_1_ (2) against BGC-823 and A2780 were 2.53 and 1.85 µM, respectively. Compound **2** showed no remarkable cytotoxic activity against HCT-8, Bel-7402 and A549 with IC_50_ > 10 µM.

## 3. Experimental

### 3.1. General

Optical rotation was measured on a Perkin-Elmer 341 digital polarimeter. The UV spectrum was taken in MeOH using a Shimadzu UV-160A, UV-Visible Recording Spectrophotometer (Shimadzu Corporation, Tokyo, Japan). Infrared spectrum was measured on a Shimadzu FTIR-8400S spectrometer. HR-ESI-MS spectrum was obtained on a LTQ Orbitrap XL mass spectrometer (Thermo Scientific, Rockford, IL USA). NMR spectra were measured on a Bruker AV-600 (600 MHz for ^1^H and 150 MHz for ^13^C) spectrometer (Bruker Biospin Inc., Rheinstetten, Germany) using CD_3_OD as solvent and tetramethylsilane (TMS) as internal standard. Silica gel (100–200 and 200–300 mesh, Qingdao Marine Chemistry Ltd., Qingdao China) and Sephadex LH-20 (20–100 µm, Pharmacia, Uppsala, Sweden) were used for column chromatography. Silica gel GF_254_ plates (Yantai Marine Chemical Co., Ltd., Yantai China) were used for thin-layer chromatography. Preparative HPLC was performed on a LUMTECH instrument with a UV detector at 210 nm and using a YMC-Pack C_18_ column (250 mm × 20 mm inside diameter (I.D.), 5 µm, YMC, Tokyo Japan).

### 3.2. Plant Material

The whole plant of *Sarcandra glabra* was collected from Jiujiang in Jiangxi Province, China, in May 2003, and authenticated by Professor Ce-ming Tan, Jiujiang Institute of Forest Plants, Jiangxi, China. A voucher specimen (No. CSH2003058018) was deposited at the Herbarium of the Institute of Medicinal Plant Development, Peking Union Medical College and Chinese Academy of Medical Sciences, Beijing, China.

### 3.3. Extraction and Isolation

The whole air-dried and powered plant material (5 kg) was exhaustively extracted three times with 70% aqueous acetone (30 L) at room temperature. The acetone extract was evaporated to dryness under reduced pressure, followed by extraction with ethyl acetate after suspension in water. The ethyl acetate fraction was separated on MCI using water and 75%, 90% MeOH–water and MeOH in sequence to afford four fractions (F1–F4). On the basis of TLC investigations, combined fraction F2 (60 g) and F3 (2.3 g) was subjected to silica gel column chromatography eluted with a gradient of petroleum ether-ethyl acetate (1:0→0:1, v/v) to afford eight fractions (Fractions A–H). Fraction G was separated on a Sephadex LH-20 column eluted with MeOH and then preparative HPLC with MeOH–H_2_O to afford **1** (3 mg), **2** (30 mg) and **3** (5 mg). Fraction E was further chromatographed on silica gel column eluted with petroleum ether: ethyl acetate: methanol (20:1:0.1, v/v/v) and followed by LH-20 (MeOH) and preparative HPLC with MeOH-H_2_O to yield compounds 4 (4 mg), 5 (6 mg), 6 (4 mg), 7 (5 mg) and 8 (2 mg). 

### 3.4. Spectral Data

Compound **1**: colorless amorphous powder. [*α*]^20^_D_ –12.6 (*c* = 0.13, MeOH). UV (MeOH) λ_max_ (log *ε*): 232 (3.49), 287 (2.92). IR (KBr) *v*_max _(cm^−1^): 3383, 2925, 2888, 1706, 1601, 1490, 1074. ESI-MS *m/z*: 413 [M + Na]^+^, 803 [2M + Na]^+^, 425 [M + Cl]^-^, 779 [2M - H] -. HR-ESI-MS: m/z 413.1192 [M + Na]+ (calcd for C20H22NaO8, 413.1212). For 1H- and 13C-NMR (CD_3_OD) spectra, see Table 1.

The structures of compounds **2**-**8** were identified by comparison of their spectral data with those reported in the literature.

### 3.5. Bioassays

The cytotoxic assays were measured on HCT-8 (human colon cancer), Bel-7402 (human liver cancer), A549 (human lung carcinoma), BGC-823 (human gastric tumor cells) and A2780 (human ovarian tumor cells) (obtained from the Institute of Materia Medica of Chinese Academy of Medical Sciences) using the MTT assay method. Cells were plated in the appropriate media on 96-well plates in a 100 µL total volume at a density of 1 × 10^5^ cell/mL. Each tumor cell line was exposed to the test compound at various concentrations in triplicates and incubated at 37 °C and 5% CO_2_ for 48 h. Cell viability was determined based on the mitochondrial conversion of MTT to formazan.

## 4. Conclusions

A new phenolic acid glycoside, benzyl 2-β-glucopyranosyloxybenzoate (**1**), together with seven known compounds, including eleutheroside B_1_ (**2**), 5-*O*-caffeoylshikimic acid (**3**), (–)-(7*S*, 8*R*)- dihydrodehydrodiconiferyl alcohol (**4**), (–)-(7*S*, 8*R*)-dihydrodehydrodiconiferyl alcohol 9-, 9′- and 4-*O*-â-D-glucopyranoside (**5**–**7**), and (–)-(7*S*, 8*R*)-5-methoxydihydrodehydrodiconiferyl alcohol 4-*O*-β-D-glucopyranoside (**8**) was isolated from the whole plant of *S. glabra*. The isolation of benzyl 2-*O*-β-D-glucopyranosyl-2-hydroxybenzoat**e** was a new addition to the molecular diversity of this species*.* Eleutheroside B_1_ (**2**) exhibited potent activity against BGC-823 and A2780 cancer cell lines with IC_50_ value of 2.53 and 1.85 µM, respectively.
